# Radiographic Correlation of Skeletal Maturation Using the Stages of Dental Calcification in a Peruvian Population

**DOI:** 10.1155/2020/4052619

**Published:** 2020-04-09

**Authors:** César Mauricio-Vilchez, Franco Mauricio, Luzmila Vilchez, Alexandra Cadenillas, Julia Medina, Frank Mayta-Tovalino

**Affiliations:** ^1^Postgraduate Department, Peruvian University Cayetano Heredia, Lima, Peru; ^2^Academic Department in Dentistry, Universidad Nacional Federico Villarreal, Lima, Peru; ^3^Postgrade Department, Faculty of Health Sciences, Universidad Científica Del Sur, Lima, Peru

## Abstract

**Objective:**

To identify the correlation between the Baccetti method of SM (skeletal maturation) and the stages of DC (dental calcification) using the Demirjian method in the Orthodontics Service of the Universidad Peruana Cayetano Heredia (UPCH) in Lima-Peru. *Materials and Methods*. The sample was obtained from panoramic and lateral radiographs of 200 subjects (116 women and 84 men) with ages between 9 and 17 years. Canine, premolar, and molar teeth calcification was evaluated using the Demirjian method while SM was evaluated by the cervical vertebrae with the Baccetti method. Spearman's correlation coefficient was used to assess the relationship between the maturation of these cervical vertebrae and DC.

**Results:**

There is a high correlation between the Baccetti method of maturation of the cervical vertebrae and the stages of DC ranging from *r* = 0.635 to 0.774 for men and from *r* = 0.677 to 0.784 for women (*p* < 0.001), the second lower molar being the one with the highest correlation (*r* = 0.774 in men) and the second lower premolar (*r* = 0.784 in women).

**Conclusions:**

Stages of DC could be used as a reliable indicator of SM.

## 1. Introduction

Skeletal maturity presents factors that can determine the growth of the human being through genetic and environmental variables. The onset of development and the speed of growth vary considerably in each person. In orthodontics and maxillary orthopedics, the analysis of maturation and the continuous observation of growth is of great importance, since, for the most part, the diagnosis and treatment plan depends on different growth factors of the human being. [1, 2].

The growth curve in humans presents different stages of acceleration and deceleration, as well as maximum growth peaks at certain ages for both sexes. For the determination of growth peaks, researchers use growth indicators, such as height, weight, voice, and menarche; however, they have been proven to be unreliable and applicative in estimating growth in the adolescent stage. More reliable studies in the area of orthodontics are the observation of bone maturation and dental age, proposed as biological indicators.

Studies have shown that the maturation of a patient's cervical vertebrae is an applicable diagnostic tool to identify the period of pubertal growth. [3, 4]. On the other hand, dental age can be determined in the dental eruption and according to the calcification stages of the teeth through panoramic radiographs, this being a reliable diagnostic element [5]. From the dental point of view, it would be ideal to know the degree of skeletal maturation, especially in patients who are in the full growth stage and need orthodontic or orthopedic treatment, which is more effective in certain stages of maturation.

Therefore, the objective of this research was to identify the radiographic correlation of SM (through the Baccetti method) using the stages of DC (through the Demirjian method) in a Peruvian population.

## 2. Methods

### 2.1. Sample Size

The present study was descriptive, transversal, retrospective, and analytical. All lateral and panoramic skull radiographs were taken from the UPCH Orthodontics Service archive in Lima, Peru. Radiographs were selected (*n* = 200). The sample size was determined according to the formula for estimating a ratio with an *α* = 0.05 and a *β* = 0.8 using the Stata® 12.0 software. Also, this research was reported following the STROBE (strengthening the reporting of observational studies in epidemiology).

### 2.2. Selection Criteria

#### 2.2.1. Inclusion Criteria

- Panoramic and lateral skull radiographs of patients between 8 and 17 years of age
- Panoramic radiographs in which the first and second premolars, first molar, canine, and second left mandibular molar are clearly observed
- Lateral radiographs of the skull in which cervical vertebrae are clearly and clearly observed
- X-rays of patients without a history of orthodontic treatment

#### 2.2.2. Exclusion Criteria

- Radiographs of patients with systemic, endocrine disorders or apparent syndromes
- Radiographs of patients with permanent teeth extracted in the left mandibular region
- Patients who have a history of premature loss of deciduous pieces
- Radiographs of patients with a history of pulp treatment in the teeth evaluated

### 2.3. Calibration

To determine the degree of reliability of the stages of DC and SM, the principal investigator was calibrated by an oral and maxillofacial radiologist specialist. The calibration was performed on digital panoramic radiographs and digital cephalometric radiographs taken with an Orthophos XG5 digital panoramic-cephalometric radiographic device (Dentsply Sirona, Erlangen, Germany). There was a previous training by the expert radiologist and subsequently the stages of dental calcification and skeletal maturation were identified by the principal investigator and the radiologist expert in the same digital radiographs. The results of the expert and the principal investigator were compared with the Kappa coefficient and the degree of concordance was 0.88 in both cases.

### 2.4. Evaluation of Radiographs

The respective coordination was carried out with the Head of the Orthodontics Service of the Stomatology Clinic of the UPCH for their permission, and then 200 panoramic and lateral radiographs were selected randomly from 9 to 17 years of both sexes, respecting the inclusion and exclusion criteria established. These radiographs were collected from the SIDEXIS XG radiological software, to be later evaluated on a computer. After being selected, only a trained observer evaluated the stages of skeletal maturation on lateral radiographs with the Baccetti method and the stages of dental calcification on panoramic radiographs with the Demirjian method.

### 2.5. Skeletal Age Evaluation

**Figure 1:**
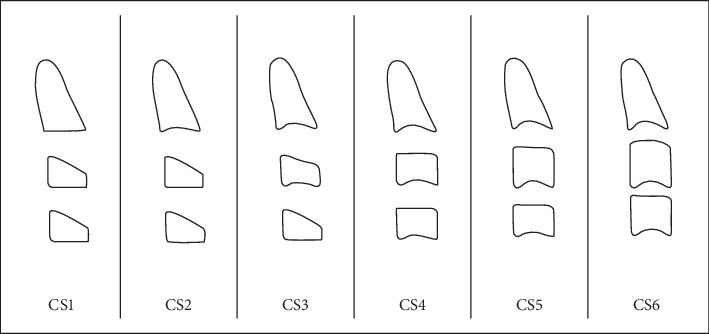
Stages of maturation of the cervical vertebrae according to Baccetti et al. [6] (2005).

The vertebrae C4, C3, and C2 were observed in detail on the lateral radiographs, and the stage presented is indicated. The interpretation was through the Baccetti method that was in 6 stages: 1, 2, 3, 4, 5, and 6 (Figure 1).

### 2.6. Evaluation of Stages of Dental Calcification

Demirjian states that there is a very high degree of symmetry between the lower teeth of the right side and left side,since he found no differences in dental age compared to the 14 lower teeth, so he used the 7 lower left teeth for analysis [5–7]. Therefore, in this investigation, only the lower left teeth were evaluated, following the Demirjian method. The process of dental calcification was observed in detail on the panoramic radiographs of the first premolar, second premolar, canine, first molar, and second left mandibular molar according to the stages of calcification by Demirjian, and the stage presented is indicated. The lower incisor was excluded because the apical closure had already formed, and the third molar was not considered in the study because it had a very low correlation with skeletal maturation [8, 9]. Dental calcification was classified according to the Demirjian method in 8 stages, A, B, C, D, E, F, G, and H. With it, the different stages of dental calcification were found and recorded on a registration form.

### 2.7. Statistical Analysis

Simple and double-entry tables were made, with their respective simple and compound bar charts. The chi-square test was used to compare the percentages. To assess the strength of the correlation between the stages of SM of cervical vertebrae and the stages of DC, the Spearman correlation coefficient with a significance level of 0.05 was calculated. All statistical analyses were performed with Stata® V12.0 software.

### 2.8. Ethical Aspects

The research was registered in the University Research Directorate with the code SIDISI 66592 and submitted to the Institutional Ethics Committee of the UPCH for approval and it was specified that there was no contact with the patients.

## 3. Results

### 3.1. Study Population

According to Table 1, the distribution of the stages of SM of cervical vertebrae according to Baccetti method varies slightly according to sex, so, in the female sex, stage V was the most frequent with 33.1% and in the male sex were stage III (29.4%) and stage IV (28.2%). When comparing them, no significant differences were found (*p* < 0.05) (Figure 1).

### 3.2. Distribution of the Stages of Skeletal Maturation of the Cervical Vertebrae

In Table 2, it is observed that the average ages increase as the stages of SM of cervical vertebrae increase according to Baccetti method, so, in stage I, the average age was 9.50 ± 0.57 in men and it was 9.0 in women, and in stage VI, the average age was 16.80 ± 0.44 in men and 16.20 ± 0.83 in women. It is also observed that the average ages for each stage of skeletal maturation were younger in the female sex (approximately 5 months) than in the male sex.

### 3.3. Distribution of Stages of Dental Calcification

In Table 3, it is observed that the most outstanding data were regarding the average ages that increase as the stages of DC increase in piece 33 according to the Demirjian method, so, in stage E, the average age was 9.00 ± 0.0 and in stage H the average age was 14.67 ± 1.74. On the other hand, it was found that the average ages increase as the stages of DC increase in piece 34 according to the Demirjian method, so, in stage E, the average age was 9.33 ± 0.52 and in stage H the average age was 15.35 ± 1.47. While the average ages are in relation to piece 37 according to the Demirjian method, stages D through H are observed, so, in stage D, the average age was 9.36 ± 0.67 and in stage H the average age was 16.47 ± 0.70.

### 3.4. Correlation of the Stages of Skeletal Maturation of the Cervical Vertebrae with the Baccetti Method with the Stages of Dental Calcification according to Demirjian Method

Table 4 shows that, in both female sex and male sex, there is a high correlation between the Baccetti method of SM of cervical vertebrae and stages of DC (*p* < 0.001), the second molar being lower than the one with the highest correlation in men (*r* = 0.774), and the second lower premolar showed the highest correlation in women (*r* = 0.784), and the first lower molar showed the lowest correlation (*r* = 0.635 in men and *r* = 0.677 in women). For boys, the sequence from the lowest to the highest was the first and second molars, first and second premolars, and canine. For women, the sequence from the lowest to the highest was the first molar, first premolar, canine, second molar, and second premolar.

## 4. Discussion

Indicators of SM and its importance in determining the peak of pubertal growth have been extensively studied [10]. Racial and ethnic differences also influence dental and skeletal maturation, especially in patients in the process of development and growth [11]. For example, different values have been found in some countries, where the use of dental and skeletal age can be used to evaluate facial growth in subjects, but it is still uncertain in Peruvian residents.

In the present investigation, the correlation between the Baccetti method of skeletal maturation and the stages of dental calcification was determined using the Demirjian method in Peruvian subjects of both sexes from 9 to 17 years of age. Some methods have been suggested to predict growth; however, many studies often use hand and wrist radiographs [1, 2, 8, 12] when the maturation stages of cervical vertebrae are known to have been better correlated with changes in skeletal growth during adolescence [3, 4]. Dental maturation is also an indicator of the biological age in children who are growing [9], using panoramic radiographs, which is a routine auxiliary examination during dental treatment. However, the time and dose of radiation exposure from hand radiography or cephalometry must be considered, which may involve some risk in children and puberty.

In the study carried out, the chronological age distributions for each stage of maturation of cervical vertebrae showed that women matured earlier than men; these results are consistent with those found by Kumar et al. [7], Mittal et al. [9], Kamal et al. [13], and Krailassiri et al. [14]. However, some studies showed that men were younger only at a stage like Uysal et al. [8] only in the Ru stage (Bjork, Grave, and Brown), Cossellu et al. [15] in stage I, and Basaran et al. [16] in stage VI. The results of the study also showed that the average ages increase as the stages of skeletal maturation of the cervical vertebrae increase, and the stages of dental calcification also increase as the age increases, except in piece 35 in the stage E which is the one with the lowest average age with 9.38 ± 0.51, and in stage D, the average age is 10.

In this study, the frequency distribution of the stages of SM according to Baccetti was evaluated with respect to each stage of DC according to Demirjian for each dental piece, resulting in the second lower molar as the dental piece with the highest degree of correlation in males, where in stage III a greater presence of stage F (72%) was observed and in stage IV a greater presence of stage G (45.8%) was observed. And in women, the dental piece with the highest degree of correlation was the second lower premolar, which in stage III showed a greater presence of stage F (83.9%), and in stage IV, the biggest percentage was present in stage G (45.2%). Baccetti et al. [6] in his method describe that the peak of mandibular growth occurs between stages III and IV, so with the results of this study, it can be noted that orthopedic treatment in men should be initiated in stage F of the lower second molar, and in women, it must begin in stage F of the second lower premolar.

The agreement between the stages of DC and SM also showed mixed results in some previous studies. For instance, Chertkow [10] found that the G stage of the lower left canine correlates with the peak of pubertal growth. Gupta et al. [17] point out that there is a significant correlation between the peak of growth and the G stage of the canine in women, but not in men, because the apical closure was already performed at the time of the pubertal growth peak. Some researchers [18, 19] indicate that stage F coincides with the onset of the adolescent growth peak, and the canine eruption in the oral cavity coincides with stage G and occurs 5 months before the peak of pubertal growth in women and 1 year before in men. Coutinho et al. [20] pointed out that the canine is in stage G, 1.3 years before the peak of pubertal growth in men and 0.4 years before in women. Krisztina et al. [21] indicated that when the Demirjian index was in stage F, then CVS was in stages 3 and 4. Krailassiri et al. [14] pointed out that the “G” stage of the second mandibular molar coincided in 39.5% with the peak of growth in women and in men the “G” stage coincided in 66.7% with the peak of growth. Uysal et al. [8] indicated that the “G” stage of the second mandibular molar coincided in 59% with the maximum growth peak in women and in men the “G” stage coincided in 60% with the growth peak. Flores-Mir et al. [22] pointed out that the “G” stage of the lower canine coincided in 92.15% with the “G” stage of Hägg and Taranger (growth peak), without finding differences in sex or nutrition.

For the second lower premolar, in males the “G” stage coincided with the maximum growth peak, in earlier stages, the dental calcification is distributed, and the “H” stage coincided with the later stages. In women, the “F” stage coincided with the growth peak, but the “F” stage also coincided in the stages before the peak. For the second lower molar in males, the “G” stage coincided with the maximum growth peak. In women, the “F” stage coincided with the peak of growth.

With respect to the correlation between Baccetti method of skeletal maturation and the stages of dental calcification, in this study, they showed high degrees of correlation for all teeth and for both sexes, with the second lower molar having the most correlation high in men (*r* = 0.7) and the second lower premolar presenting the biggest correlation in women (*r* = 0.7), and the first lower molar evidenced the lowest correlation (*r* = 0.6 in men and *r* = 0.6 in women).

Previous publications determined the degree of correlation between dental calcification and skeletal maturation finding diverse values [22–26]. Uysal et al. [8] in Turkish subjects indicated that the lower second molar showed the greatest correlation (*r* = 0.82 in women and *r* = 0.70 in men), while the lower third molar showed the lowest correlation (*r* = 0.49 in women and *r* = 0.41 in men). On the other hand, Cericato et al. [27] evaluated Brazilian subjects and found that the second lower left premolar showed the biggest correlation and the first lower molar showed the lowest correlation. Otherwise, Mittal et al. [9] indicated that in Indian subjects the second molar showed the highest correlation (*r* = 0.75 in men and *r* = 0.81 in women) and the third molar showed the lowest correlation (*r* = 0.40 in men and *r* = 0.41 in women). Another example is the study by Rozylo-Kalinowska et al. [28] in Polish subjects, who indicated that the lower second premolar showed the highest correlation in women (*r* = 0.59) and the lower canine showed the highest correlation in men (*r* = 0.52). Also, Basaran et al. [16] indicated that in Turkish settlers the lower second molar showed the highest correlation (*r* = 0.84 in women and *r* = 0.91 in men) and the third molar showed the lowest correlation (*r* = 0.42 in women and *r* = 0.47 in men). Finally, Krailassiri et al. [14] mentioned that in Thai settlers the second lower premolar showed the highest correlation (*r* = 0.69 in women and *r* = 0.66 in men).

The contribution of this study, as a clinical application, indicates that orthopedic treatment in male patients should be initiated at the stage of calcification F of the lower second molar and, in female patients, should be initiated at the stage of calcification F of the second lower premolar. The correlation found between the Baccetti method and the stages of DC using the Demirjian method is highly significant; with these results, we confirm that the SM of a patient could be estimated with the help of panoramic radiography by evaluating stages of DC.

A weakness of this study was the lack of order of a database by age and gender of the patients, with their respective radiographs in the archive of the Postgraduate Orthodontics Service and in the Sidexis XG Radiological Software, which generated a difficulty in looking for radiographs according to the inclusion criteria of the study, having a limitation in expanding the sample size.

The importance of this research lies in the fact that it established knowledge to the Peruvian population about how differences in age and sex, as well as the state of health of people, influence the stages of bone maturation and the stages of mineralization of the teeth. The time and speed of ossification are primarily affected by the socioeconomic development of the Peruvian population. In this way, in the future, it will prevent the appearance of bruxism and other parafunctions of the temporomandibular joint [29]. Of all the studies and procedures used for the evaluation of the stages of bone maturation and dental mineralization, the ones that have reached the most diffusion have been the qualitative-descriptive methods; this general method has the purpose of predicting and establishing the moment specific to the different states or events of sudden pubertal growth.

Another great contribution of this study is that SM presents genetic and environmental variables that can alter growth. The beginning and peak of growth vary considerably. Some girls are usually the first to mature with short periods of growth, while others mature late with periods of prolonged growth. Bone age is a reliable indicator in relation to chronological age. That is why in order to define the stage of SM, a series of indicators have been proposed; in this investigation, the correlation between the Baccetti method of cervical vertebrae maturation and the stages of DC using the Demirjian method was evaluated.

Finally, for the clinical justification, according to the results, the patient's growth phase can be determined, and the growth indicators can be used in the identification of the stages of bone maturation and dental mineralization; if there is a correlation between both methods, we can determine the maximum peak of growth of a patient by observing a panoramic radiograph, thus facilitating the clinician in the diagnosis.

## 5. Conclusions

Within the limitations of this research, it is concluded that the distribution of the stages of skeletal maturation of the cervical vertebrae according to the Baccetti method varies according to sex, but when contrasting them, no significant differences were found. On the other hand, the average ages increase as the skeletal maturation stages of the cervical vertebrae increase according to the Baccetti method. Finally, a correlation was found between the skeletal maturation of the cervical vertebrae with the stages of dental calcification for all teeth and for both sexes.

## Figures and Tables

**Table 1:** Distribution of the stages of skeletal maturation of the cervical vertebrae with the Baccetti method according to sex.

Baccetti	Female		Male		Total
	*n*	%	*n*	%	
I	6	5.2	4	4.8	10
II	4	3.4	6	7	10
III	31	27	25	29.4	56
IV	31	27	24	28.2	55
V	38	33.1	21	24.7	59
VI	5	4.3	5	5.9	10
Total	115	100.0	85	100.0	200

Chi-square test: *p*=0.848.

**Table 2:** Distribution of the stages of skeletal maturation of the cervical vertebrae with the Baccetti method according to age.

Baccetti	Gender	*n*	Mean	SD
I	Male	4	9.50	0.57
	Female	6	9.00	0

II	Male	6	9.83	0.98
	Female	4	9.50	1.00

III	Male	25	11.08	0.99
	Female	31	10.41	0.92

IV	Male	24	13.70	1.75
	Female	31	13.22	1.47

V	Male	21	15.04	1.28
	Female	38	14.63	1.65

VI	Male	5	16.80	0.44
	Female	5	16.20	0.83

Total		200		

**Table 3:** Distribution of stages of dental calcification with the Demirjian method according to age.

Tooth piece	*n*	Mean	SD	Min	Max
Tooth 33					
E	3	9.00	0.00	9	9
F	60	10.43	1.41	9	17
G	40	12.20	1.32	9	15
H	97	14.67	1.74	10	17

Tooth 34					
E	6	9.33	0.52	9	10
F	71	10.54	1.33	9	17
G	60	13.22	1.26	11	16
H	63	15.35	1.47	11	17

Tooth 35					
D	1	10.00	0.00	10	10
E	13	9.38	0.51	9	10
F	84	11.10	1.46	9	17
G	49	13.84	1.30	11	17
H	53	15.51	1.41	11	17

Tooth 36					
G	75	10.68	1.57	9	17
H	125	14.10	1.96	10	17

Tooth 37					
D	11	9.36	0.67	9	11
E	31	10.03	1.17	9	14
F	67	11.87	1.39	10	17
G	72	14.47	1.42	11	17
H	19	16.47	0.70	15	17

Total	200	12.82	2.47	9	17

**Table 4:** Correlation between the Baccetti method of skeletal maturation of the cervical vertebrae and the stages of dental calcification using the Demirjian method according to sex.

Tooth piece	Male		Female	
	*r*	*p*	*r*	*p*
Tooth 33	0.761	0.001	0.779	0.001
Tooth 34	0.763	0.001	0.777	0.001
Tooth 35	0.650	0.001	0.784	0.001
Tooth 36	0.635	0.001	0.677	0.001
Tooth 37	0.774	0.001	0.782	0.001

Spearman's correlation coefficient. Level of significance: *p* < 0.05.

## Data Availability

The applied data in the statistical analysis of this research will be available upon authorization of the corresponding managers of the university.

## References

[1] Fishman L. S. (1979). Chronological versus skeletal age, an evaluation of craniofacial growth. *Angle Orthodontist*.

[2] Fishman L. S. (1982). Radiographic evaluation of skeletal maturation. A clinically oriented method based on hand-wrist films. *Angle Orthodontist*.

[3] Franchi L., Baccetti T., McNamara J. A. (2003). The cervical vertebral maturation method: some need for clarification. *American Journal of Orthodontics and Dentofacial Orthopedics*.

[4] Hassel B., Farman A. (1995). Skeletal maturation evaluation using cervical vertebrae. *American Journal of Orthodontics and Dentofacial Orthopedics*.

[5] Demirjian A., Goldstein H., Tanner J. M. (1973). A new system for dental age assessment. *Humam Biololgy*.

[6] Baccetti T., Franchi L., McNamara J. A. (2005). The cervical vertebral maturation (CVM) method for the assessment of optimal treatment timing in dentofacial orthopedics. *Seminars in Orthodontics*.

[7] Kumar S., Singla A., Sharma R., Virdi M. S., Anupam A., Mittal B. (2012). Skeletal maturation evaluation using mandibular second molar calcification stages. *The Angle Orthodontist*.

[8] Uysal T., Sari Z., Ramoglu S., Basciftci F. (2004). Relationships between dental and skeletal maturity in Turkish subjects. *Angle Orthodontics*.

[9] Mittal S., Singla A., Virdi M., Sharma R., Mittal B. (2011). Co-relation between determination of skeletal maturation using cervical vertebrae and dental calcification stages. *Internet Journal of Forensic Science*.

[10] Chertkow S. (1980). Tooth mineralization as an indicator of the pubertal growth spurt. *American Journal of Orthodontics*.

[11] Hägg U., Taranger J. (1982). Maturation indicators and the pubertal growth spurt. *American Journal of Orthodontics*.

[12] Monirifard M M., Sichani A. V., Sichani A. V., Yaraghi N. (2017). Correlation of dental age and cervical vertebral maturation and chronological age in an Iranian population based on radiography. *International Journal of Orthodontics*.

[13] Kamal A. T., Shaikh A., Fida M. (2018). Assessment of skeletal maturity using the calcification stages of permanent mandibular teeth. *Dental Press Journal of Orthodontics*.

[14] Krailassiri S., Anuwongnukroh N., Dechkunakorn S. (2002). Relationships between dental calcification stages and skeletal maturity indicators in Thai individuals. *Angle Orthodontics*.

[15] Cossellu G., Biagi R., Pisani L., Barbieri V., Farronato G. (2014). Relationship between mandibular second molar calcification and cervical vertebrae maturity in Italian children and young adult. *European Journal of Paediatric Dentistry*.

[16] Basaran G., Ozer T., Hamamci N. (2007). Cervical vertebral and dental maturity in Turkish subjects. *American Journal of Orthodontics and Dentofacial Orthopedics*.

[17] Gupta S., Chada M., Sharma A. (1995). Assessment of puberty growth spurt in boys and girls: a dental radiographic method. *Journal of Indian Society of Pedodontics and Preventive Dentistry*.

[18] Demirjian A., Levesque G.-Y. (1980). Sexual differences in dental development and prediction of emergence. *Journal of Dental Research*.

[19] Fernandes-Retto P., Matos D., Ferreira M., Bugaighis I., Delgado A. (2019). Cervical vertebral maturation and its relationship to circum-pubertal phases of the dentition in a cohort of Portuguese individuals. *Journal of Clinical and Experimental Dentistry*.

[20] Coutinho S., Buschang P. H., Miranda F. (1993). Relationships between mandibular canine calcification stages and skeletal maturity. *American Journal of Orthodontics and Dentofacial Orthopedics*.

[21] Krisztina M. I., Ogodescu A., Réka G., Zsuzsa B. (2013). Evaluation of the skeletal maturation using lower first premolar mineralisation. *Acta Medica Marisiensis*.

[22] Flores-Mir C., Mauricio F., Orellana M., Major P. (2005). Association between growth stunting with dental development and skeletal maturation stage. *Angle Orthodontics*.

[23] Mollabashi V., Yousefi F., Gharebabaei L., Amini P. (2019). The relation between dental age and cervical vertebral maturation in orthodontic patients aged 8 to 16 years: a cross-sectional study. *International Orthodontics*.

[24] Ojha A., Prasanth M., Singh V., Sihag T., Bhati V., Tomar H. (2018). Assessment of correlation between dental calcification stages and skeletal maturity indicators. *Journal of Forensic Dental Sciences*.

[25] Felemban N. H. (2017). Correlation between cervical vertebral maturation stages and dental maturation in a Saudi sample. *Acta Stomatologica Croatica*.

[26] Macha M., Lamba B., Avula J. S. S., Muthineni S., Margana P. G. J. S., Chitoori P. (2017). Estimation of correlation between chronological age, skeletal age and dental age in children—a cross-sectional study. *Journal of Clinical and Diagnostic Research*.

[27] Cericato G. O., Franco A., Bittencourt M. A. V., Nunes M. A. P., Paranhos L. R. (2016). Correlating skeletal and dental developmental stages using radiographic parameters. *Journal of Forensic and Legal Medicine*.

[28] Rozylo-Kalinowska I., Kolasa-Raczka A., Kalinowski P. (2011). Relationship between dental age according to Demirjian and cervical vertebrae maturity in Polish children. *The European Journal of Orthodontics*.

[29] Marín M., Rodríguez Y., Gamboa E., Ríos J., Rosas J., Mayta-Tovalino F. (2019). Level of work stress and factors associated with bruxism in the military crew of the Peruvian air force. *Medical Journal Armed Forces India*.

